# Utilization of patient-reported outcome measures in shoulder and elbow surgery: a survey-based study

**DOI:** 10.1016/j.xrrt.2025.07.015

**Published:** 2025-08-11

**Authors:** Niclas Lutz, Nils Käßer, Rony-Orijit Dey Hazra, Doruk Akgün, Markus Scheibel, Benedikt Schliemann, David Alexander Back

**Affiliations:** a Center for Musculoskeletal Surgery, Charité-Universitätsmedizin Berlin, Berlin, Germany; b Freie Universität Berlin, Humboldt-Universität zu Berlin, Berlin, Germany; c Berlin Institute of Health, Berlin, Germany; d Department for Shoulder and Elbow Surgery, Schulthess Clinic, Zurich, Switzerland; e Department of Trauma and Orthopaedic Surgery, FRANZISKUS Center of Shoulder, Elbow and Hand Surgery, Münster, Germany

**Keywords:** PROMs, Evaluation, Digital, Shoulder, Elbow, Physician

## Abstract

**Background:**

Patient-reported outcome measures (PROMs) are widely established in shoulder and elbow surgery, as they are able to reflect the functional outcome after a respective treatment. However, various challenges exist to add PROMs into in the daily clinical practice. The aim of the presented study was to analyze the utilization of PROMs among shoulder and elbow physicians in Germany, Austria, and Switzerland.

**Methods:**

An online questionnaire (SurveyMonkey) assessed the use of shoulder- and elbow-specific PROMs in orthopedic and trauma surgery clinics across the D-A-CH region. Distributed via the D-A-CH Society for Shoulder and Elbow Surgery email list, surveys were enlisted from April to December 2024. Participation was voluntary, anonymous, and implied consent. Due to anonymity, European data protection regulations were not applicable. Ethical approval was obtained.

**Results:**

Among 124 participants, 82% were male and 17% female. While 54% did not use PROMs, 46% incorporated them into daily practice. For shoulders, Disabilities of the Arm, Shoulder and Hand Questionnaire (DASH)/quick Disabilities of the Arm, Shoulder and Hand Questionnaire (73%), Simple Shoulder Test (29%), and ROWE Score (21%) were most frequently used. Regarding elbows, 54% did not use PROMs, DASH (29%) and Mayo Elbow Performance Score (25%) were most common among users. PROMs were primarily collected via paper questionnaires (58%) or not with digital tools (23%). PROMs were valued for scientific research (73%), therapy monitoring (71%), and treatment quality improvement (56%). Key disadvantages included subjective patient perception (71%), needed patient collaboration (67%), and additional workload (63%).

**Conclusion:**

PROMs are still rarely used in daily clinical practice but are valued for enhancing research and treatment quality by integrating patients' subjective experiences. Key barriers include the lack of affordable, user-friendly digital solutions. Future efforts may focus on professional society recommendations for suitable PROMs and the establishment of their digital measuring.

In the recent decade, patient-reported outcome measures (PROMs) have increasingly come into focus of research and clinical practice, as they capture the subjective perspective of patients and contribute to a more comprehensive assessment of treatment success.^5^ Especially in rural areas, the remote collection of PROMs could enhance patients' monitoring.^13^ PROMs are standardized questionnaires that allow patients' experiences regarding pain, functional limitations, and overall quality of life to be documented. These patient-centered measurement tools provide valuable insights into the individual recovery process and help tailor treatment and recovery to patients' personal needs and goals.^25^ Different studies have shown that PROMs such as the Disabilities of the Arm, Shoulder, and Hand Questionnaire (DASH), American Shoulder and Elbow Surgeons (ASES) score, Shoulder Pain and Disability Index and many other scores are frequently used to evaluate treatment outcomes in shoulder and elbow injuries.^26^^,^^15^

This is of great interest, since in value-based health care, patient benefit is considered the central measure of treatment success.^9^ Michael E. Porter defined value in health care as being determined by the outcomes achieved, rather than the volume of services provided.^28^ PROMs could help to acknowledge patient perspectives, quantify their experiences in real time, and integrate these results into clinical decision-making.^20^ This can help to better adapt treatment strategies to patients' needs and promote their engagement in the treatment process, ultimately leading to improved satisfaction and outcomes.^11^

However, integrating PROMs into clinical practice also presents challenges. These include selecting appropriate instruments that are both reliable and sensitive to changes and tailored to the specific patient population.^22^ The practical implementation in clinical practice also poses a hurdle, particularly in busy environments, where collecting and analyzing PROMs data require additional effort.^24^ Despite these challenges, PROMs offer significant benefits, such as improved communication between patients and health-care providers, as well as better alignment of treatment strategies with individual expectations and needs.^25^

To advance the widespread adoption of PROMs, there is a need for simple and cost-effective data collection methods. Ongoing digitalization in medicine offers numerous opportunities in this regard. Web-based options, such as digital remote postoperative PROM measurement, have the potential to reduce costs and staffing requirements by minimizing in-person visits.^34^ They can also enable more seamless monitoring of the healing and rehabilitation process compared to traditional follow-up regimens, making it possible for health-care professionals to intervene, if necessary.^30^^,^^33^

The aim of this study was to analyze the use of PROMs in orthopedic clinics specializing in shoulder and elbow surgery across the German-speaking European countries—Austria, Germany, and Switzerland—with a particular focus on digital vs. analog data collection methods, as well as the identification of key benefits, barriers, and facilitators.

## Methods

### Study design

An online-based questionnaire was created using the SurveyMonkey tool (SurveyMonkey Europe UC, Dublin, Ireland) to examine the use of shoulder- and elbow-specific PROMs in orthopedic and trauma surgery clinics and medical practices throughout the D-A-CH region (encompassing the the German-speaking European countries of Austria, Germany and Switzerland). The questionnaire was distributed via the email list of the D-A-CH Society for Shoulder and Elbow Surgery (DVSE) and was open for participation from April to December 2024. All participants were informed in an introductory letter, preceding the questionnaire, about its purpose and about data collection and processing procedures. Participation in the survey was voluntary and anonymous; completion of the questionnaire was thus considered as consent. Due to the anonymous nature of the survey, European data protection regulations did not apply. The ethics committee of the Charité – Universitätsmedizin Berlin gave its approval (No.: EA4/144/24).

### Structure of the questionnaire

**Figure 1:**
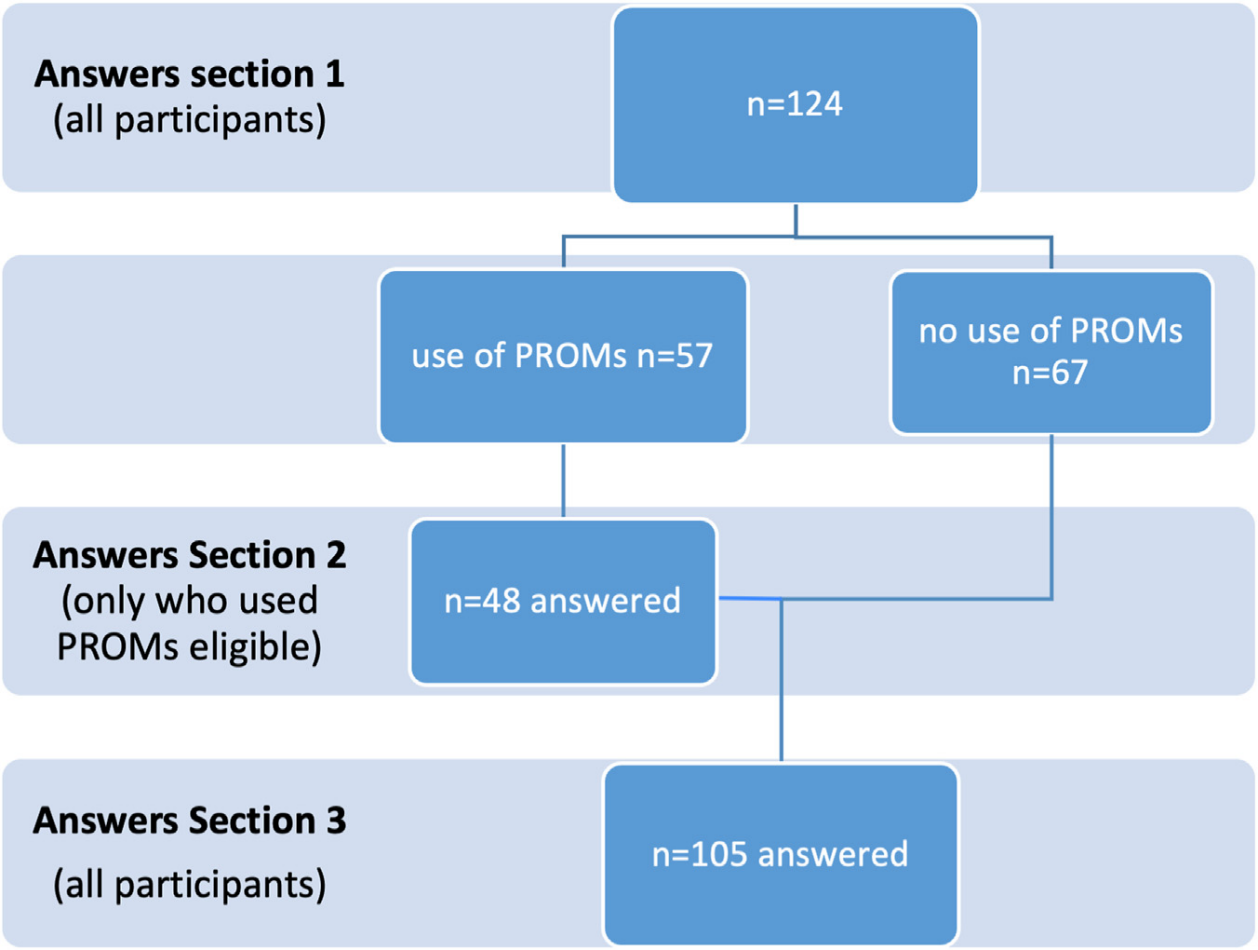
Number of participants that answered each of the three sections of the questionnaire. *PROMs*, patient-reported outcome measures.

An initial version of the questionnaire was revised and finalized by 3 experts in the field of shoulder and elbow surgery. The result consisted of a total of 19 questions (8 single-choice, 10 multiple-choice, 1 open-ended), with an option for additional open-ended responses in 13 of the single- or multiple-choice questions. These questions were divided into 3 sections (Fig. 1).

• Part 1: Demographics and general use of PROMs

In this section, demographic data on the participants were collected, including gender and position in their respective health-care facility. In addition, participants were asked whether they use PROMs for quality assurance at all. Depending on the answer to this question, participants were then directed either to a special section of the questionnaire (Part 2, see below) or a general section (Part 3, see below). All 19 questions could only be answered if the third question was answered “Yes.” Otherwise, participants were only able to answer 10 out of 19 possible questions.

• Part 2: Specific questions on the PROMs used and their collection

This part was only presented to those participants who had indicated to use PROMs. Participants were asked which PROMs they used from a given table with the option to add free-text responses. They were also asked how long they have been collecting these PROMs, the methods of data collection (digital, by mail, by telephone, etc.), whether a specific software was used, if PROMs were collected in both surgical and conservative treatments, and whether introducing PROMs has led to adjustments in clinical practice (eg, postoperative procedures, etc.).

• Part 3: Opinions on the use of PROMs in clinical practice

The final 7 questions explored the respondents' overall opinions on PROMs—whether they generally considered the use of PROMs to be worthwhile, perceived advantages and disadvantages, and specific views regarding digital PROM collection. Finally, participants were asked whether they would like to receive a recommendation from a medical society regarding the use of specific PROMs.

### Data processing and statistics

The sample size was based on the assumption that *P* = 40% of participants used PROMs. Assuming a response rate of 10%, we anticipated n = 120 evaluable questionnaires. A one-sided binomial test would then have a power of over 80% to reject the null hypothesis H0: *P* ≤ .27 at a one-sided significance level of alpha = 0.025. Here, p represents the proportion of specialists who use PROMs. The sample size calculation was performed using R version 4.2.1. The questionnaire data were analyzed descriptively by reporting both relative and absolute frequencies of all item responses. A 2-sided 95% confidence interval was calculated for the proportion of respondents who use PROMs.

## Results

Out of 1,280 members in the DVSE email list, 124 colleagues answered the online based questionnaire (response rate of 9.7%).

### Demographics and general use of PROMs

The first section of the questionnaire was completed by a total of 124 participants, of which 82.3% (n = 102) were male, 16.9% (n = 21) female, and 0.8% (n = 1) diverse.

Regarding the positions, 31.5% (n = 39) were senior physicians, 20.2% (n = 25) were self-employed in a private practice or medical care center, 15.3% (n = 19) served as head of departments, and 11.3% (n = 14) were residents in a hospital, 10.5% (n = 13) were employed specialists in a hospital, 5.7% (n = 7) were students, and 3.2% (n = 4) were employed specialists in outpatient care. The remaining 2.4% (n = 3) chose “other,” which included 1 research assistant, 1 senior physician, and 1 medical director in a medical care center.

Asked whether they used PROMs for quality assurance in their orthopedic clinic or practice, 54% (n = 67) indicated “No,” and 46% (n = 57) “Yes.”

### Specific questions on the PROMs used and their collection

The second section could only be answered by those n = 57 of 124 participants who had indicated to use PROMs. n = 48 of 57 answered all questions of section 2, n = 9 of 57 did not answer the questions. The percentages mentioned in this section refer to the 48 participants who answered the questions (n = 48 = 100%).

**Figure 2:**
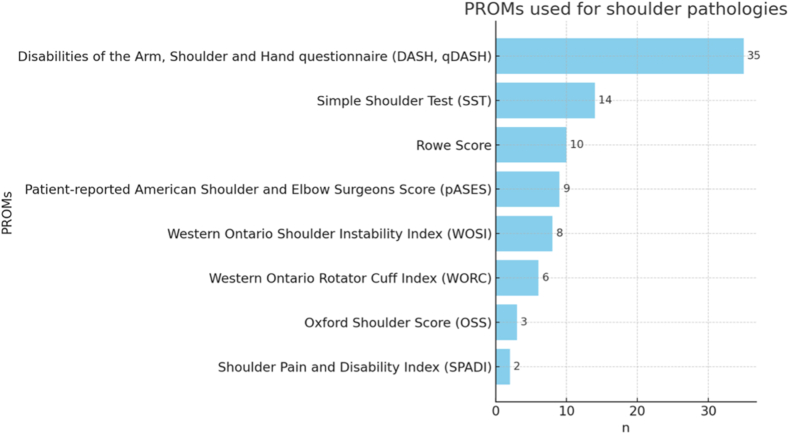
PROMs used for shoulder pathologies (multiple answers possible). *PROMs*, patient-reported outcome measures.

The PROMs used in the treatment of shoulder pathologies as indicated by the participants are listed in Figure 2. Five participants (n = 5 of 48) did not use PROMs for shoulder pathologies. Of the 13 (n = 13 of 48) who indicated the use of other PROMs not listed in the table, 5 participants mentioned the Constant Score, 3 the Subjective Shoulder Value, 2 the ASES without the patient-reported component, 2 the European Quality of Life 5 Dimensions, 1 the Mayo Elbow Performance Index (which is elbow-specific), and 1 (n = 1 of 48) a visual analog scale without further description.

**Figure 3:**
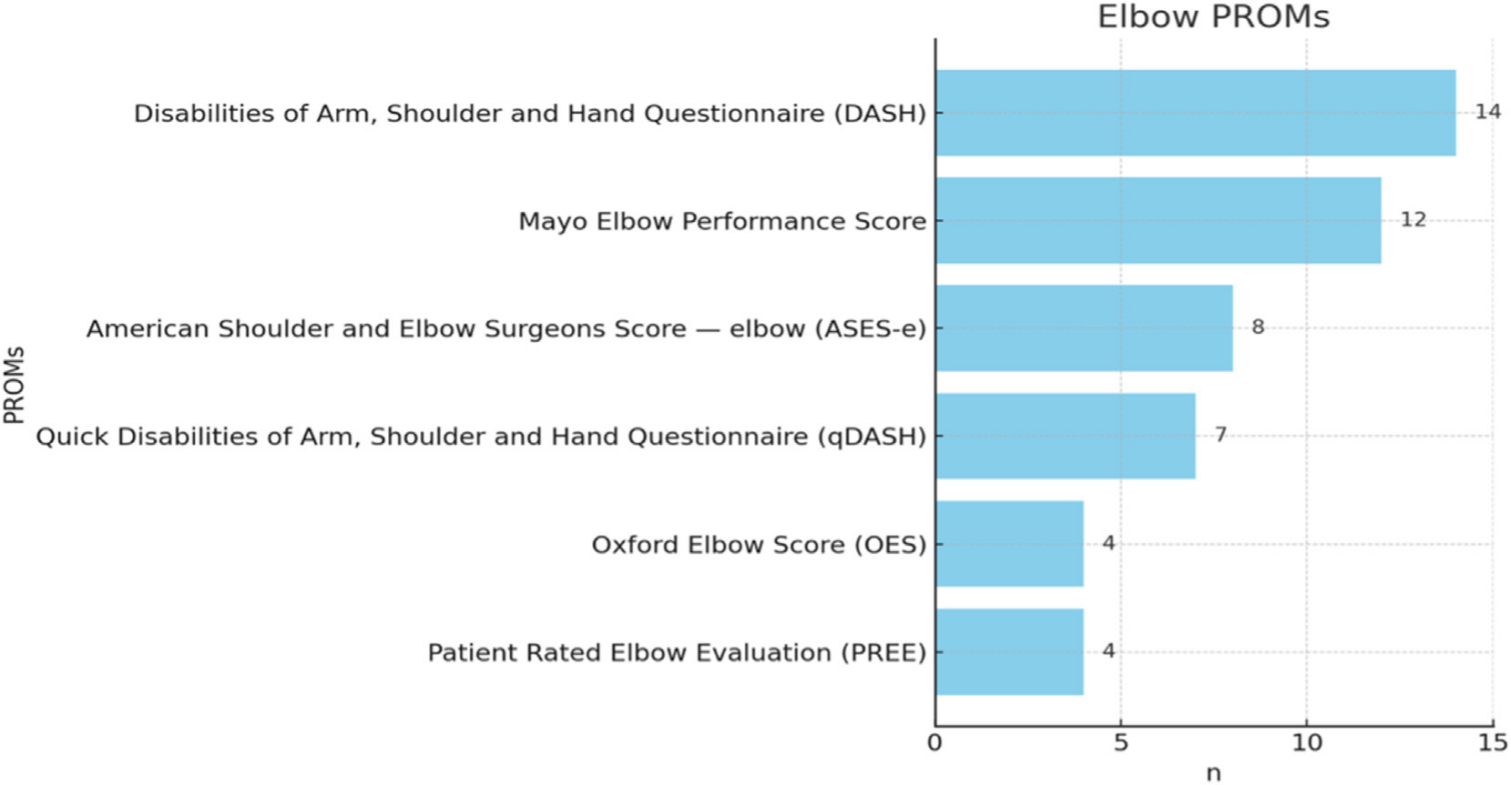
PROMs used for elbow pathologies (multiple answers possible). *PROMs*, patient-reported outcome measures.

Participants' selections for elbow-specific PROMs are shown in Figure 3.

n = 26 of 48 respondents (54.2%), despite collecting PROMs, did not use any for elbow pathologies. One participant stated as free answer for another elbow-specific PROM the Mayo Elbow Performance Index which is essentially the same as the given Mayo Elbow Performance Score.

Regarding the duration of PROM usage, 37.5% (n = 18 of 48) indicated that they had been collecting PROMs for more than 5 years, 25% (n = 12 of 48) between 2 and 5 years, 16.7% 12.5% (n = 6 of 48) for 1-2 years, 8.3% (n = 4 of 48) for between 6 months and 1 year and 16,67% (n = 8 of 48) for less than 6 months at the time of the survey.

As collection mode, 58.3% of the participants (n = 28 of 48) stated they used paper questionnaires for collecting PROMs, 22.9% (n = 11 of 48) used a digital tool that is not integrated into their clinic or practice software, 20.8% (n = 10 of 48) used a digital approach integrated into their hospital information system or practice software, 18.8% (n = 9 of 48) collected PROMs by mail, 12.5% (n = 6 of 48) by telephone, and 12.5% (n = 6 of 48) opted for some other collection method. Among these, 2 (n = 2 of 48) mentioned the German-Language Arthroscopy Register (DART), and 1 respondent mentioned a register (not further specified).

In terms of digital PROMs collection, indicated software solutions were as follows:

- Heartbeat Medical (HRTBT Medical Solutions GmbH, Berlin, Germany) (n = 3 of 48)
- REDCap (Vanderbilt University, Nashville, USA) (n = 3 of 48)
- Nelly Solutions (Nelly Solutions GmbH, Berlin, Germany) (n = 1 of 48),
- Sana Software (Sana Commerce EMEA B.V., Rotterdam, Netherlands) (n = 1 of 48)
- DART (DART GmbH, Neuss, Germany) (n = 1 of 48)

Further answers were SPSS (IBM Deutschland GmbH, Böblingen, Germany) (n = 1 of 48) and 3 unspecified solutions.

Regarding the timing of data collection for surgically treated patients, 66.7% (n = 32 of 48) reported that they collected PROMs at fixed postoperative time points. A further 39.6% (n = 19 of 48) also collected them preoperatively as part of surgical planning, 22.9% (n = 11 of 48) at every outpatient visit, 16.7% (n = 8 of 48) at the time of the first consultation of initial contact and 10.4% (n = 5 of 48) upon hospital admission. Three respondents (n = 3 of 48) specified the exact postoperative intervals in free-text answers with “6 weeks 3, 6, and 12 months postoperatively”, “6 and 12 months postoperatively”, “according to the underlying condition, typically during the final examination or after 1 year in cases of endoprosthesis”.

For nonoperatively treated patients, 66.7% (n = 32 of 48) did not collect PROMs. Others 16.7% (n = 8/48) collected PROMs at every patient visit or following the last appointment 16.7% (n = 8 of 48). Two collected PROMs at the first consultation, and 1 did so upon hospital admission. One free-text answer indicated the collection of PROMs only when conservative treatment had failed.

**Figure 4:**
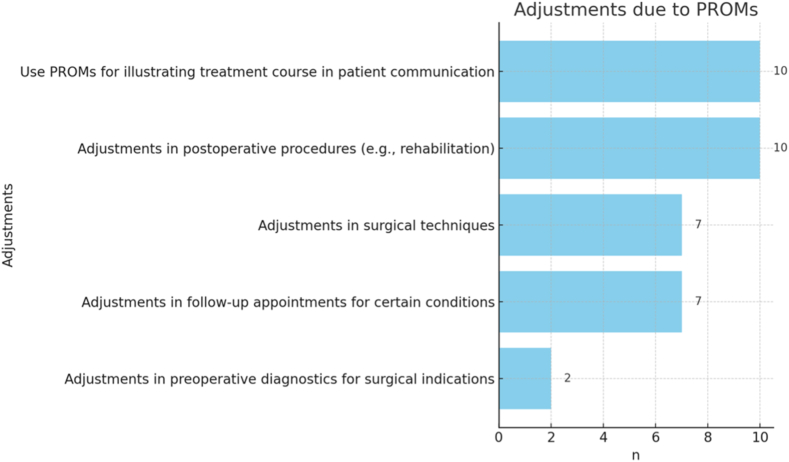
Process adjustments resulting from the introduction of PROMs. *PROMs*, patient-reported outcome measures.

Figure 4 shows the adjustments in clinical practice due to the introduction of PROMs. Of 48, 58.3% (n = 28) of the participants indicated no adjustments at all. Two participants used the free text answer and stated the implementation of modifications in regard to analgetic administration (n = 1 of 48) and the use of the Constant Score as an indicator for surgery (n = 1 of 48).

When asked about response rates, 18.8% (n = 9 of 49) estimated the range between 76%-100%, 27.1% (n = 13 of 48) between 51%-75%. 37.5% (n = 18 of 48) between 26%-50% and 16.7% (n = 8 of 48) reported a response rate of 0%-25%.

### Opinions on the use of Patient-Reported Outcome Measures in clinical practice

The third section of the questionnaire was again open to all participants. Out of 124, n = 105 of 124 answered the questions. The percentages mentioned in this section refer to the 105 participants who answered the questions (n = 105 = 100%).

Asked if they considered it reasonable for PROMs to be collected on a mandatory basis due to quality assurance, 61.9% (n = 65 of 105) agreed and 37.1% (n = 39 of 105) disagreed. Twenty-four free-text responses were recorded of which n = 2 were inconclusive. Supporters emphasized improved quality of care (n = 4), benefits for scientific studies (n = 2), better comparability among clinics (n = 2) and increased credibility of treatment outcomes by incorporating patient perspectives (n = 2). Opponents primarily cited the added bureaucratic burden (n = 7), concerns that mandatory PROM collection would merely generate vast amounts of minimally informative data (n = 3), insufficient compensation for the additional effort (n = 2) and the currently still insufficient standardization of PROMs (n = 1).

**Figure 5:**
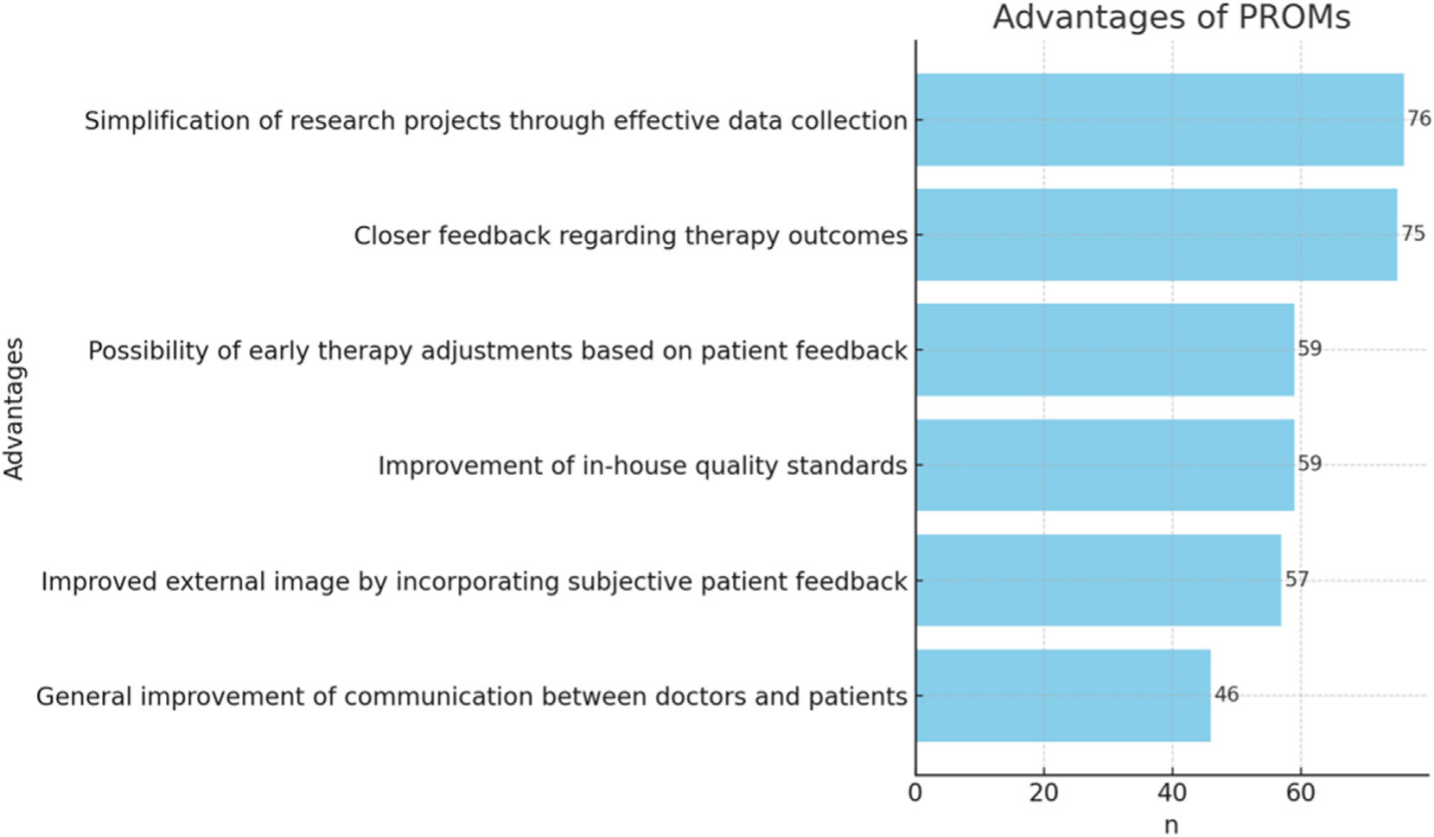
General advantages of PROMs according to participants. *PROMs*, patient-reported outcome measures.

Regarding general advantages of PROM collection (Fig. 5), The responses highlight that participants primarily view PROM collection as a tool to facilitate research and enhance clinical insight. The most commonly recognized advantages include the simplification of research projects through structured data collection and improved feedback on therapy outcomes. Additionally, PROMs were perceived to support early therapeutic adjustments and raise both internal quality standards and external credibility by integrating patients' subjective experiences. Only a small minority saw no added value or expressed skepticism, suggesting a generally positive attitude toward PROM implementation.

**Figure 6:**
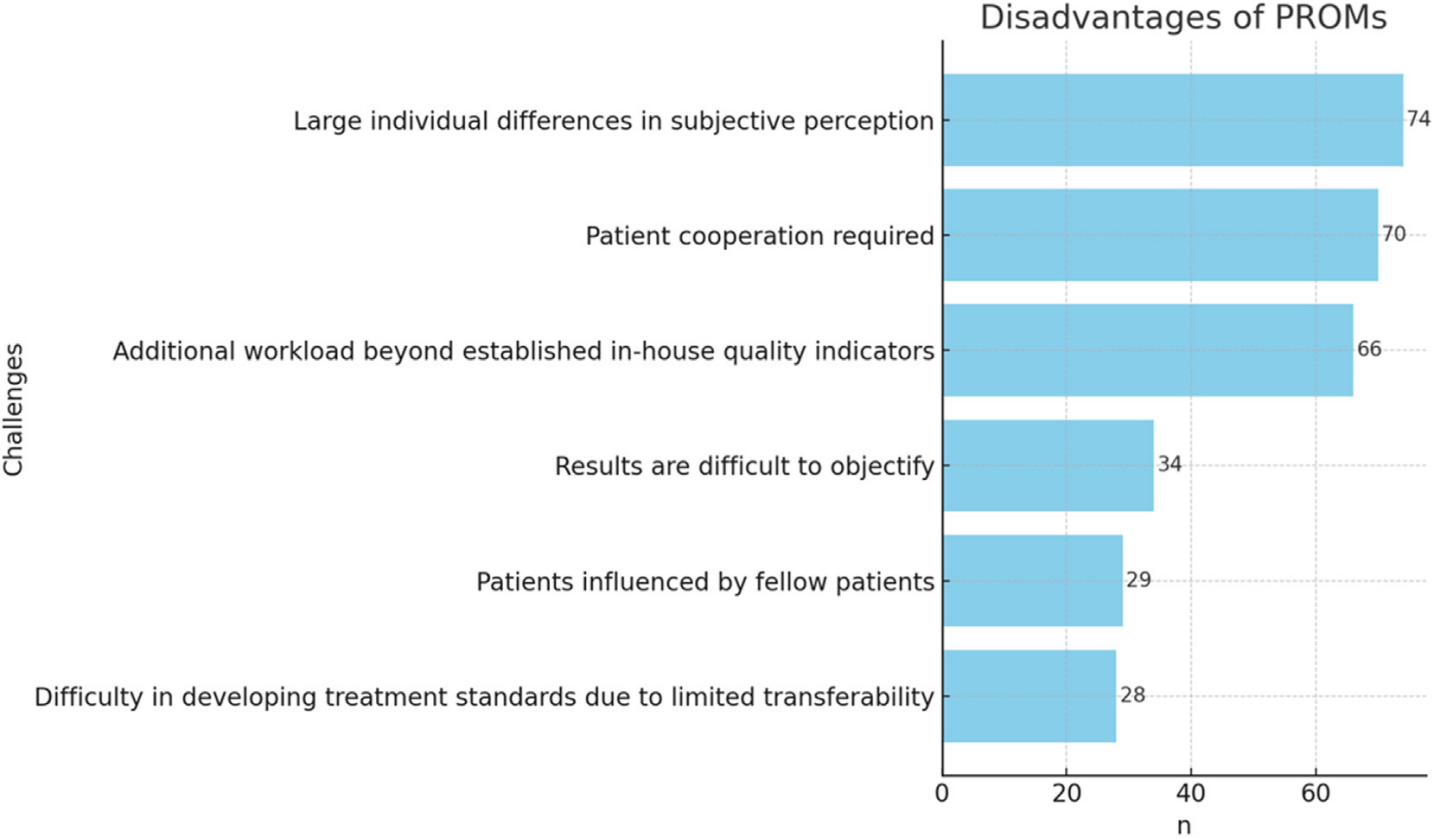
General disadvantages of PROMs according to participants. *PROMs*, patient-reported outcome measures.

Figure 6 shows the opinions on general disadvantages of PROMs. Five participants (n = 5 of 105) emphasized the increased workload under scarce resources, the difficulties in making the results truly objective and problems with the needed cooperation of the patients to receive meaningful data.

Regarding the advantages of digital PROM collection, 72.4% (n = 76 of 105) cited the simplicity and efficiency of sending questionnaires by email or SMS at predefined intervals. Of 105, 69.5% (n = 73) mentioned that digital systems can securely store results and allow easy retrieval, and 59.1% (n = 62 of 105) would see time savings because patients would not need to return for in-person visits. For 42.2% (n = 43 of 105), digital tools would require no extra staff. One free-text answer found digital collection promising but noted that the response rate would need to be evaluated.

Looking at the disadvantages of digital PROM collection, 81% (n = 85 of 105) mentioned the more stringent data protection requirements when using external tools to gather and store patient data and 60% (n = 63 of 105) were concerned about additional software costs. Another 41.9% (n = 44 of 105) felt that digital methods were too impersonal and difficult to control, potentially reducing patient motivation. Of 105, 11.4% (n = 12) added comments pointing to the need for patients' technical know-how (particularly for older individuals), required access to email or SMS and the lack of objective monitoring parameters (eg x-rays, range of motion).

Of 105, 71.4% (n = 75) of the participants supported to welcome a recommendation from the DVSE on digital PROM collection, while 28.6% (n = 30 of 105) would not.

With the final voluntary question, the participants who did not yet use PROMs where asked for their reasons. Twenty-five participants (n = 25 of 124) completed the question. n = 22 of 25 answers could be categorized in 5 main groups, n = 3 of 25 answers were inconclusive.

- The additional time and staffing burden (n = 10 of 25)
- High costs and integration problems (n = 5 of 25)
- PROMs are not yet widely established or standardized (n = 4 of 25)
- Lack of management support (n = 1 of 25)
- Insufficient software options (n = 2 of 25)

## Discussion

The relevance of PROMs is steadily increasing in shoulder and elbow surgery. There are now more than 30 PROMs available for assessing patients' subjective experiences; however, their validation varies, and they are not yet used uniformly.^15^ Hence, the goal of this study was to provide an overview of the current state of PROMs used in the field of shoulder and elbow surgery in the German-speaking European countries, as well as the methods of their collection in the respective health-care institutions. The most important finding was that more than half of the respondents did not use PROMs in their clinical practice. When PROMs were used, nearly 60% of participants still collected PROMs using paper questionnaires. Nevertheless, participants saw potential in the digitalization of PROM collection, enabling easier patient communication and follow-up (eg, via email or SMS), while saving personnel and resources by reducing the need for frequent in-person visits.

The responses to the questionnaire indicate that PROMs are not yet widely used in shoulder and elbow surgery in the D-A-CH region. Similar findings have been reported in previous studies. In the orthopedic community of Saudi Arabia, a study published in 2020 showed that 69.1% of 262 orthopedic surgeons did not use PROMs in their daily routines.^2^^,^^19^ A systematic review that screened 6 mayor orthopedic journals from 2011 to 2019 for PROM use found that PROMs were only used in 29% of orthopedic trauma articles.^2^^,^^19^ For both shoulder and elbow pathologies, the participants in this survey most frequently used the DASH or quick Disabilities of the Arm, Shoulder and Hand Questionnaire. However, a large-scale systematic review on PROMs published in 2021 with 506 studies and 36,553 patients, showed that internationally the ASES was most used in scientific studies, followed by the DASH and SPADI, while the DASH was available in most languages, indicating a high level of international acceptance.^25^ It was noticeable, that while using PROMs, more than half of the participants (n = 26 of 48) did not use PROMs for elbow pathologies while only 5 (n = 5 of 48) did not use shoulder PROMs.

The need for better digitalization of PROM collection is evident from the high proportion of paper-based methods, as shown in this study. The use of paper questionnaires is associated with high costs, among other factors. For instance, the National Health Service in the UK is reported to bear annual costs of approximately £800,000 for analog PROMs collection.^29^

According to the participants in this study, the main advantages of a well-developed digital PROMs system include secure data storage and saving time and resources by reducing the need for frequent in-person follow-ups. Similar findings are reflected in the literature. The disadvantages, such as more complex data protection requirements and additional software costs, were also frequently mentioned.^23^ Regardless of the method of collection and storage, most respondents saw general advantages of PROMs in improving the comparability of scientific studies and providing closer feedback regarding therapy outcomes, which goes along with observations of other studies.^12^^,^^16^^,^^18^^,^^27^ Moreover, the standardization of PROMs to enhance the comparability of scientific studies, such as through the introduction and further development of the Patient-Reported Outcomes Measurement Information System, is a frequently discussed topic.^32^ Organizational challenges in implementing PROMs in clinical practice were primarily attributed to the additional workload in an already high-stress work environment and the need for patient collaboration. Participants identified potential qualitative issues in the suspected differences in subjective patient perceptions. Given the already high workload in the medical field, it is unsurprising that the additional effort required to collect PROMs is viewed critically.^3^^,^^14^ To overcome the organizational challenges associated with PROM implementation, the need for a coordinator in a leadership role has been repeatedly emphasized in studies.^3^^,^^4^ This would meet the high acceptance found in this study for recommendations from the professional society regarding the selection of suitable PROMs, also since the large number of PROMs can make it difficult for medical professionals to choose appropriate outcome measures throughout different medical fields.^1^^,^^7^^,^^31^

Although the European Shoulder and Elbow Society (SECEC/ESSSE) has recommended using the Constant Score in all peer-reviewed studies since 1992,^8^ it was not included as a selectable option in this study, as it was not regarded as PROM due to its dependence on a clinical examination.^17^

This study has several limitations that should be considered when interpreting the data. First, out of 1,280 members of the DVSE that were reached via email, 124 members completed the questionnaire, making it difficult to represent a comprehensive and representative opinion of the professional society. However, similar response rates have been reported in comparable online surveys.^10^ Additionally, there is a possibility that colleagues who hold a generally negative view of PROMs and do not see their implementation as beneficial may have opted not to participate in a voluntary online survey. This could lead to an underrepresentation of their views among the participants, potentially introducing bias.

In the future, professional societies can play an important role in recommending PROMs and supporting cost-effective digital solutions to improve quality of care. Beyond, newer digital approaches such as *Ecological Momentary Computerised Adaptive Testing* should be further examined for their use to closer track patients' subjective perspective.^6^ For adding patient-centered objectivity, big data generation and its analysis with approaches like *Bring Your Own Device* with the use of patients' mobile phones or wearables for everyday use can be an interesting combination with PROMs,^21^ including also artificial intelligence based algorithms.

## Conclusion

The utilization of PROMs is increasingly seen as beneficial for holistic patient care by incorporating subjective patient experiences as a quality indicator. Despite this growing interest, integrating PROMs into routine clinical practice faces challenges such as a lack of affordable, user-friendly digital tools and uncertainty about which PROMs are most suitable for specific conditions to allow for comparable treatment outcomes. The survey emphasizes the need for professional societies to issue specific recommendations for PROMs for everyday treatment of patients and promote cost-effective digital solutions to fully realize the potential of PROMs in improving treatment quality.

## Disclaimers

Funding: The study did not receive any funding. It was supported by the German, Austrian and Swiss Shoulder and Elbow Society (DVSE).

Conflicts of interest: The authors, their immediate families, and any research foundations with which they are affiliated have not received any financial payments or other benefits from any commercial entity related to the subject of this article.
